# Human TOP1 residues implicated in species specificity of HIV-1 infection are required for interaction with BTBD2, and RNAi of BTBD2 in old world monkey and human cells increases permissiveness to HIV-1 infection

**DOI:** 10.1186/1743-422X-7-332

**Published:** 2010-11-20

**Authors:** Bharat Khurana, Lei Zhuang, Prasun K Moitra, Tzanko S Stantchev, Christopher C Broder, Mary Lou Cutler, Peter D'Arpa

**Affiliations:** 1 Department of Pathology, Uniformed Services University of the Health Sciences, 4301 Jones Bridge Road, Bethesda, MD (20814) USA; 2 Department of Biochemistry and Molecular Biology, Uniformed Services University of the Health Sciences, 4301 Jones Bridge Road, Bethesda, MD (20814) USA; 3 Department of Microbiology and Immunology, Uniformed Services University of the Health Sciences, 4301 Jones Bridge Road, Bethesda, MD (20814) USA; 4 United States Military Cancer Institute, Uniformed Services University of the Health Sciences, 4301 Jones Bridge Road, Bethesda, MD (20814) USA

## Abstract

**Background:**

Host determinants of HIV-1 viral tropism include factors from producer cells that affect the efficiency of productive infection and factors in target cells that block infection after viral entry. TRIM5α restricts HIV-1 infection at an early post-entry step through a mechanism associated with rapid disassembly of the retroviral capsid. Topoisomerase I (TOP1) appears to play a role in HIV-1 viral tropism by incorporating into or otherwise modulating virions affecting the efficiency of a post-entry step, as the expression of human TOP1 in African Green Monkey (AGM) virion-producing cells increased the infectivity of progeny virions by five-fold. This infectivity enhancement required human TOP1 residues 236 and 237 as their replacement with the AGM counterpart residues abolished the infectivity enhancement. Our previous studies showed that TOP1 interacts with BTBD1 and BTBD2, two proteins which co-localize with the TRIM5α splice variant TRIM5δ in cytoplasmic bodies. Because BTBD1 and BTBD2 interact with one HIV-1 viral tropism factor, TOP1, and co-localize with a splice variant of another, we investigated the potential involvement of BTBD1 and BTBD2 in HIV-1 restriction.

**Results:**

We show that the interaction of BTBD1 and BTBD2 with TOP1 requires *hu*-TOP1 residues 236 and 237, the same residues required to enhance the infectivity of progeny virions when *hu*-TOP1 is expressed in AGM producer cells. Additionally, interference with the expression of BTBD2 in AGM and human 293T target cells increased their permissiveness to HIV-1 infection two- to three-fold.

**Conclusions:**

These results do not exclude the possibility that BTBD2 may modestly restrict HIV-1 infection via colocation with TRIM5 variants in cytoplasmic bodies.

## Background

Upon entry into target cells, retroviruses undergo several transformations to establish a productive infection which include uncoating of the viral core, reverse transcription, nuclear access, and integration of the viral DNA into the host genome [1,2]. Factor(s) incorporated into HIV-1 virions from producer cells and factor(s) present in target cells determine viral tropism [3-10].

Topoisomerase I (TOP1) activity has been found to be associated with HIV virions [11], and the species of TOP1 expressed in virion producer cells has been reported to significantly influence viral infectivity: HIV-1 virions produced by African Green Monkey (AGM) cells were 85-90% less infective to human cells as compared to virions produced by human cells [7]. Shoya et al. reported that expression of human-TOP1, but not AGM-TOP1, in HIV-1-producing AGM cells increased the infectivity of progeny virions about five-fold [7]. This enhancement to the infectivity of HIV-1 virions provided by the expression of *hu*-TOP1 in AGM cells was dependent on *hu*-TOP1 residues E236 and N237, as replacement of these residues with their AGM counterparts abolished the activity enhancement. The infectivity enhancement was associated with a four-fold greater copy number of HIV-1 DNA in target cells [7]. In contrast to Old World monkey producer cells, in human producer cells (293T) the expression of *hu*-TOP1 only slightly increased viral infectivity. Also, expression of AGM TOP1, or *hu*-TOP1 with residues 236 and 237 replaced with the AGM counterpart residues (i.e., E236D/N237S), in human producer cells caused virions to have four-fold less infectivity [7].

TRIM5α is a major factor that restricts HIV-1 infection of Old World monkey cells, and expression of rhesus monkey TRIM5α in human cells confers potent resistance to HIV-1 infection [8]. Conversely, interference with TRIM5α expression in Old World monkey cells relieves the block to HIV-1 infection [8]. The TRIM family of proteins contains a tripartite motif that includes RING, B-box 2 and coiled-coil (cc) domains. Many TRIM proteins, including TRIM5α, assemble into cytoplasmic structures [12]. We previously reported that a non-restricting splice variant of TRIM5, TRIM5δ, localizes to cytoplasmic bodies together with BTBD1 and BTBD2. BTBD1 and BTBD2 proteins interact with TOP1, share 80% amino acid sequence identity with each other, and contain a BTB/POZ domain and kelch-like and PHR-like regions [13,14]. The BTBD/POZ domain mediates homo- and hetero-dimerization and some BTB domains bind the Cul3 ubiquitin ligase and select substrates for ubiquitylation [15-18]. The kelch repeat is a β-propeller structure that appears in numerous proteins as a protein-protein interaction site. Our observations that the BTBD1 and BTBD2 proteins physically interact with one HIV-1 restriction factor, TOP1, and co-localize with a splice variant of TRIM5α prompted us to investigate the potential involvement of BTBD1 and BTBD2 in restricting HIV infection.

Here we show that the same two *hu*-TOP1 residues required for the enhancement of the infectivity of progeny virions when *hu*-TOP1 is expressed in AGM producer cells are also required for *hu*-TOP1 to bind BTBD1 and BTBD2. We also show that interference with the expression of BTBD2 modestly increases HIV-1 infection in both non-permissive AGM cells and permissive 293T cells. These two observations together suggest that BTBD2 may be linked to restriction of HIV-1 infectivity.

## Methods

### Cell Culture

African green monkey (COS-1), human adenocarcinoma HeLa, and large T antigen expressing human embryonic kidney (293T) cell lines were maintained in Dulbeco's MEM (DMEM) supplemented with 2 mM L-Glutamine, 10% fetal bovine serum, and penicillin/streptomycin.

### TOP1 constructs, yeast two-hybrid and GST-pulldown

**Figure 1:**
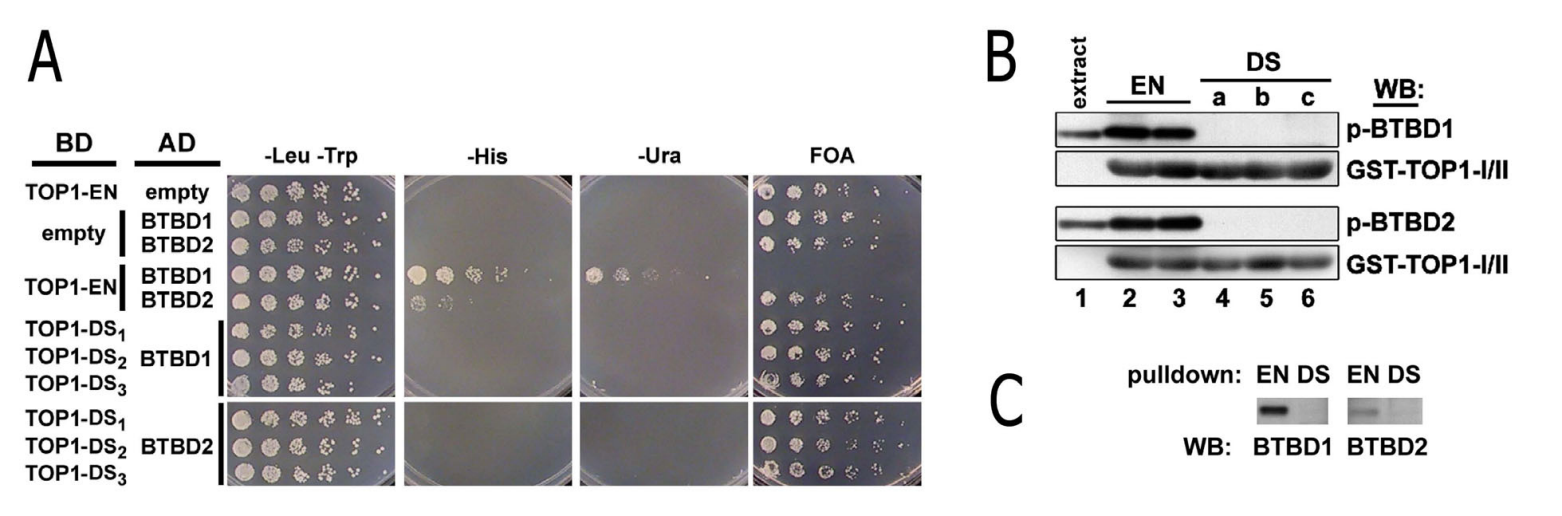
**Two-hybrid and co-precipitation assays show the requirement for *hu*-TOP1 residues E236/N237 for binding BTBD1 and BTBD2**. ***A***. The Proquest two hybrid system (Invitrogen) was used with clones as previously described [14]. The TOP1 cap domain [20] (aa 219-433) with either human residues E236/N237 (TOP1-EN) or AGM residues D236/S237 (TOP1-DS; three independent clones designated subscript 1, 2 and 3) fused to the Gal4 DNA binding domain (BD) were expressed from the minus Leu selectable plasmid. BTBD1 (aa 82-525) and BTBD2 (aa 1-482) were expressed as Gal4 activating domain (AD) fusions from the minus Trp selectable plasmid. Yeast transformed with these plasmids or with their parent plasmids without TOP1, BTBD1 or BTBD2 sequences (empty) were replica plated using three fold dilutions onto plates without Leu and Trp (-Leu,-Trp) to detect the presence of the plasmids. To detect the two hybrid protein interactions, the yeast were replica plated onto plates lacking Leu, Trp and His (and with 25 mM 3AT to adjust His selection stringency); plates lacking Leu, Trp and Ura (-Ura); or, plates lacking Leu and Trp and containing 5-fluoorotic acid (FOA, a substrate of URA3 that is converted into a toxic compound). *hu*-TOP1_E236/N237 _(TOP1-EN) shows interaction with BTBD1 and BTBD2 but three clones of *hu*-TOP1_D236/S237 _do not. ***B***. GST-pulldown assay: GST fused to the cap domain of *hu*-TOP1 (EN, duplicate lanes; DS, triplicate clones) on glutathione beads were incubated with cell extracts containing C-terminal regions of BTBD1 (aa 186-482) and BTBD2 (aa 219-525). After incubation and washing, the bead pellets were processed for Western blots using polyclonal antibody recognizing both BTBD1 and BTBD2. GST-capTOP1_E236/N237 _co-precipitates BTBD1 (p-BTBD1) and BTBD2 (p-BTBD2) but GST-capTOP1_D236/S237 _does not. Immunostaining of GST-capTOP1 is with GST antibody (GST-TOP1-I/II). Lane 1 was loaded with 1/30^th ^of the extract containing BTBD1 and BTBD2. ***C***. As in "B" but endogenous BTBD1 and BTBD2 enriched from 293T cells is detected using peptide antibodies specific to each protein that were pulled down from extracts of 293T cells by GST-capTOP1_E236/N237 _but not by GST-capTOP1_D236/S237_. All experimental results were reproduced in at least two replicate experiments.

The amino terminus of *hu*-TOP1 was fused to the Tap tag [19], producing TAP-TOP_E236/N237 _or TAP-TOP_D236/S237_. These plasmids were transiently transfected into HeLa cells, and the expressed TAP-TOP1 proteins were purified and used to determine that the amino acid replacements had no effect on DNA relaxation activity (Data not shown). Two hybrid and GST-pulldown assays were performed as previously described and in the legend to Figure 1[14].

### Immunofluorescence

Cells on coverslips were fixed with 4% paraformaldehyde for 10 min on ice and treated with 0.1% Triton X-100 for 10 minutes on ice. Fixed cells were blocked with PBS containing 3% BSA for 1 hour at room temperature before incubation with primary antibodies. Secondary antibody incubations were at room temperature for 45 min. For detection of cytoplasmic bodies, cells were reacted with rabbit anti-BTBD1 peptide antibody [13] followed by Alexa Fluor 568 goat anti-rabbit IgG (Molecular Probes). Coverslips were mounted using Prolong Antifade kit (Invitrogen, Carlsbad CA). Fluorescence images were obtained using an Olympus IX70 inverted fluorescence microscope equipped with a Princeton Instrument cooled CCD camera integrated with Scanalytics IP-Lab software. For quantification of BTBD1/D2 cytoplasmic bodies, the acquired images were analyzed using TIFFany 3 (Caffeine Software) and the cytoplasmic bodies were quantified using Mathematica software (Wolfram research).

### BTBD1 and BTBD2 shRNA constructs

The pSIREN-RetroQ vector system (Clontech, Palo Alto CA) was used to produce BTBD1 and BTBD2 specific short hairpin shRNA (under the control of the human U6 promoter). Target sequences within both BTBD1 (NM_025238) and BTBD2 (NM_017797) sequences were identified and verified by BLAST analysis for their specificity to BTBD1 and BTBD2, respectively. The pSIREN-BTBD1 (D1#3) shRNA vector contained the BTBD1-specific target sequence CACTCAAAGGTCCAGATTC, and the pSIREN-BTBD2 (D2#7) shRNA vector contained the BTBD2-specific target sequence AGGTCATCTTCTACACCTA. Oligonucleotides used to construct the negative control (S-ve) and luciferase (S-luc) shRNA vectors were supplied by the manufacturer. ShRNA vectors were constructed by inserting the annealed oligonucleotides into the linearized pSIREN-RetroQ vector. The nucleotide sequence of all clones was determined to confirm the expected insertions.

### Silencing of BTBD1, BTBD2 and TRIM5α genes

Control (S-ve and S-luc), BTBD1 (D1#3) and BTBD2 (D2#7) shRNA constructs (16 μg) were transfected into 2 × 10^6 ^COS-1 cells per 10 cm dish using Lipofectamine-2000 transfection reagent (Invitrogen, Carlsbad CA) at a DNA (μg) to Lipofectamine™ 2000 (μl) ratio of 1:2.5. After 48 h to 72 h, cells expressing the shRNA constructs were harvested, tested for reduced expression of BTBD1 and BTBD2 by Northern blotting, and reseeded prior to HIV-1-luc infection.

For silencing TRIM5α, small-interfering RNA (siRNA) directed against TRIM5α, 5'-GCCUUACGAAGUCUGAAACUU-3' [8], was used. A non-specific control siRNA, 5'-AUGAACGUGAAUUGCUCAAUU-3' [8], was included in the experiments. Both TRIM5α-specific and non-specific control siRNAs were purchased from Dharmacon RNA Technologies (Lafayette, CO). COS-1 cells (1 × 10^6^) were transfected in 100-mm dishes with 15 nM siRNA and 30 μl of Lipofectamine RNAiMAX (Invitrogen, Carlsbad CA). After 48 h to 72 h, cells were harvested, tested for reduced expression of TRIM5α mRNA by Northern blotting, and reseeded prior to HIV-1-luc infection.

### RNA isolation and Northern blotting

Cells were lysed with 1 ml of TriPure Isolation Reagent (Roche, Indianapolis IN) and RNA was purified. Equal amounts of total RNA (10-20 μg) were loaded for electrophoresis on a 1% formaldehyde-agarose gel and transferred to a nylon membrane. The membranes were subsequently hybridized with gene specific, ^32^P-labeled, random primed probes, and the amount of mRNA was quantified on a beta counter.

### Western Blot

Cells were lysed in buffer A (50 mM Tris, pH 8.0, 150 mM NaCl, 1.0% Nonidet P-40, 0.5% deoxycholate) and protein concentrations were determined using BCA kit (Pierce, Rockford IL). Equivalent amounts of protein were resolved in Tris-glycine gels, transferred to PVDF membrane, and probed with antibody generated against full length BTBD2 that recognizes both BTBD1 and BTBD2 (Figure 1B), peptides specific for BTBD1 or BTBD2 that do not cross-react (Figure 1C), *hu*-TOP1 (rabbit F14; LAE Biotechnology Co., Ltd., Rockville MD) or GST. The blots were developed using the appropriate horseradish peroxidase-conjugated secondary antibodies and ECL reagent (Amersham, GE Health, Piscataway NJ).

### Reporter gene virus and Infection assays

Vesicular stomatitis virus (VSV) G glycoprotein pseudotyped retrovirus particles containing a luciferase reporter gene were produced by cotransfecting 293T cells with the HIV-1 derived backbone plasmid (pNL4-3 env^- ^Vpr^+ ^Luc) and a plasmid for VSV-G expression (pCG). COS-1 cells transfected with shRNA constructs, siRNAs or TOP1 expression constructs were seeded in 48-well plates (20,000 cells/well), incubated for 48 hours and subsequently inoculated with different amounts of VSV-G pseudotyped, HIV-1 luciferase reporter gene encoding virus (VSV-G/HIV-1/Luc). Cells were lysed 48 h post-infection (0.1% Triton-X-100 in PBS) and the virus infectivity was evaluated by measuring the luciferase activity of the cell lysates using the Luciferase Assay Kit (Promega, Madison WI) with a Fluoroskan Ascent luminometer (Thermo Labsystems).

## Results

### E236 and E237 of TOP1 are required for interaction with BTBD1 and BTBD2

In a previous study we used two hybrid and pulldown assays together with deletion fragment mapping to demonstrate the requirement for TOP1 residues 215 to 329 (15% of the 765 aa protein) for the physical interaction with BTBD1 and BTBD2, two proteins which co-localize with TRIM5δ [14]. This 115 amino acid fragment of TOP1 contains residues E236 and N237 previously shown by Shoya et al. to be key determinants of species-specific tropism of HIV infection. We therefore tested the requirement of these TOP1 residues for interaction with BTBD1 and BTBD2. We compared the *hu*-TOP1 core domain [20] to the core domain with residues E236 and N237 replaced with the AGM counterpart residues (E236D/N237S) for ability to interact with BTBD1 and BTBD2 using two-hybrid and GST-pulldown assays. Replacement of *hu*-TOP1 residues E236/N237 with AGM residues D236/S237 abolished the two-hybrid interactions (Figure 1A). For the pull-down assay, we fused GST to an independently folding domain of *hu*-TOP1 termed the cap domain that consists of core subdomains 1 and 2 and includes E236/N237 (GST-*hu*-TOP1_cap_). This fusion protein co-precipitated purified BTBD1 and BTBD2 fragments, but GST-*hu*-TOP1_cap _with E236D/N237S failed to co-precipitate BTBD1 or BTBD2 (Figure 1B).

Next we tested whether GST-*hu*-TOP1_cap _could co-precipitate endogenous BTBD1 and BTBD2 from HeLa cell extracts. Our previous efforts to detect these proteins on Western blots of whole cell lysates using antibodies generated to major portions of the proteins or to unique peptides were not successful, suggesting limited amounts of the proteins in the cells. To test whether GST-*hu*-TOP1_cap _could enrich for and co-precipitate endogenous BTBD1 and BTBD2 cellular proteins, we incubated HeLa cell extracts with GST-*hu*-TOP1_cap _or GST-*hu*-TOP1_cap _having E236/N237 replaced with AGM residues D236/S237. GST-*hu*-TOP1_cap _co-precipitated proteins of about 60 kDa, which were recognized by antibodies specific to BTBD1 or BTBD2, respectively, and were not co-precipitated by GST-*hu*-TOP1_cap _with AGM residues D236/S237 (Figure 1C). Thus, the two residues of TOP1 required for enhancing the infectivity of progeny virions [7] were also required for TOP1 to physically interact with cellular BTBD1 and BTBD2.

### Tranfection of BTBD1 and BTBD2 shRNA silencing plasmids decreases target mRNAs and cytoplasmic bodies detected with BTBD1-specific antibody

**Figure 2:**
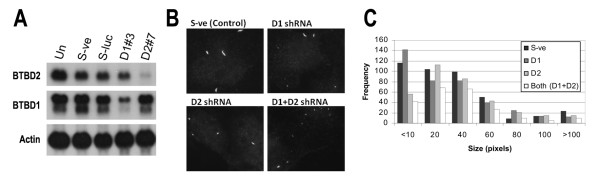
**COS-1 cells transfected with plasmids encoding shRNAs specific to BTBD1 and BTBD2 show reductions in the specific mRNAs and cytoplasmic bodies stained with BTBD1-specific antibodies**. ***A***. Northern blot shows reduced levels of expression of BTBD1 and BTBD2 mRNA in COS-1 cells transfected with BTBD1 or BTBD2 specific silencer constructs. COS-1 cells were untransfected (Un) or transfected with the negative control vector, pSIREN (S-ve), the pSIREN vector encoding Luciferase silencer (S-luc), pSIREN vector encoding BTBD1 shRNA (D1 #3) or BTBD2 shRNA (D2 #7). 48 hours post-transfection, transiently transfected and untransfected (COS-1) cells were harvested for RNA isolation. The Northern blot was hybridized with BTBD1 and BTBD2 specific probes. Equal amounts of RNA (10 μg) were loaded onto each lane as seen with the β-actin specific probe. ***B***. Representative immunofluorescence images showing cytoplasmic bodies in COS-1 cells transfected with pSIREN negative control vector (S-ve), silencer constructs for BTBD1 or BTBD2, or both BTBD1 and BTBD2. Seventy-two hours post-transfection cells were labeled with rabbit BTBD1-specific peptide antibody (#4358) followed by Alexa Fluor 568 goat anti-rabbit IgG (Molecular Probes). ***C***. Quantification of BTBD1/D2 cytoplasmic bodies. The acquired images were analyzed using TIFFany 3 (Caffeine Software) and the cytoplasmic bodies were quantified using Mathematica software (Wolfram research).

We investigated the potential involvement of BTBD1 and BTBD2 in restricting HIV-1 infection based on the observations that: 1) BTBD1 and BTBD2 co-localize with TRIM5δ [13], a splice variant of TRIM5α; 2) *hu*-TOP1 residues E236/N237 are required for the infectivity enhancement provided to virions by the expression of *hu*-TOP1 in AGM virion-producer cells; and, 3) these same human residues are required for the physical interaction of *hu*-TOP1 with BTBD1 and BTBD2. We depleted BTBD1 or BTBD2 using RNAi. Plasmids expressing short hairpin RNA specific for BTBD1 or BTBD2 and control RNA were transfected in COS-1 African green monkey kidney cells. Northern blotting showed a 70-80% reduction of BTBD1 and BTBD2 mRNA in cells transfected with specific shRNA for BTBD1 or BTBD2, respectively, as compared to untransfected or vector controls (Figure 2A).

To evaluate the effect of RNA interference of BTBD1 and BTBD2 on their protein levels, we quantified cytoplasmic bodies stained with BTBD1-specific antibody in COS-1 cells 72 hours after transfection of the silencer plasmids. In cells transfected with only one specific silencer shRNA construct, either against BTBD1 or BTBD2, the average number of cytoplasmic bodies per cell was similar to control cells. In cells transfected with both shRNA silencer plasmids, the average number of cytoplasmic bodies per cell was reduced by 40%, from ~3.8 to 2.2 (Table 1 and Figures 2B and 2C).

**Table 1:** shRNA interference of both BTBD1 and BTBD2 reduces the number of cytoplasmic bodies

	S-ve*	sh-BTBD1	sh-BTBD2	sh-BTBD1 + sh-BTBD2
**TOTAL cytoplasmic bodies**	413	395	348	227
**Total cells counted**	110	104	103	105
**Cytoplasmic bodies/cell**	3.8	3.8	3.4	2.2

* vector control

### Transfection of BTBD2 shRNA silencing plasmid increases permissiveness to HIV-1 infection

**Figure 3:**
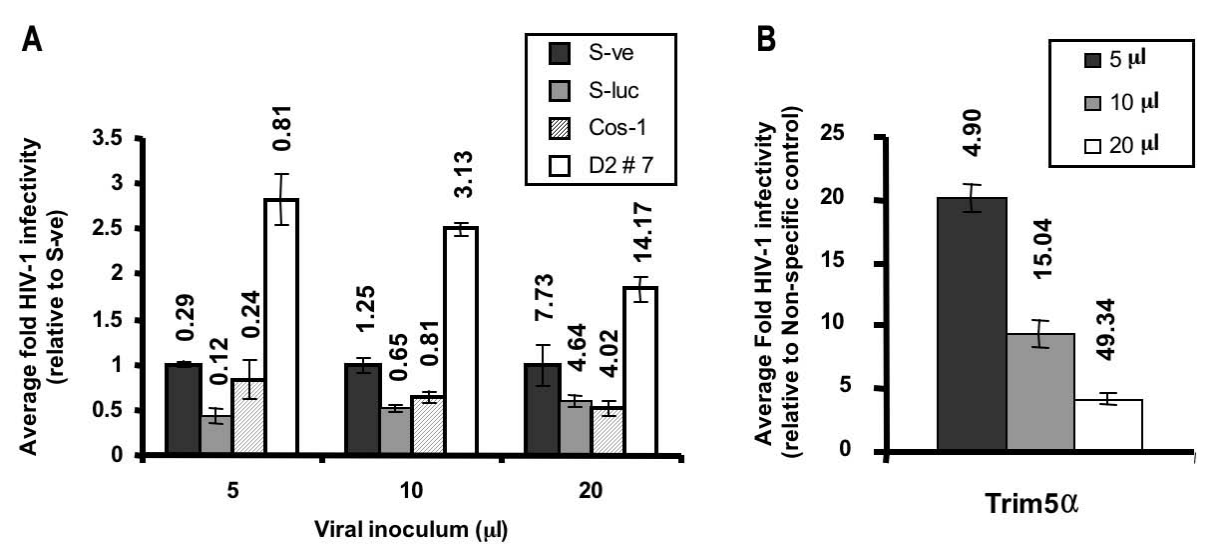
**Permissiveness to HIV-1 infectivity increases in COS-1 cells transfected with BTBD2 silencer construct**. ***A***. COS-1 cells transfected with silencer plasmids or controls were subsequently infected with VSV-G/HIV-1/Luc (p24 titer: 440 ng/ml): Uninfected COS-1 cells (Cos1), pSIREN control (S-ve), pSIREN encoding Luciferase shRNA (S-luc), pSIREN encoding BTBD2 shRNA (D2 #7). Bold numbers above bars represent actual average infectivity. ***B***. HIV-1 infectivity in COS-1 cells transfected with either non-specific siRNA or TRIM5α specific siRNA. Bold numbers above bars represent actual average infectivity data. Forty-eight hours post-transfection, transiently transfected and untransfected (COS-1) cells were seeded onto 48-well plates at 20,000 cells/well) and inoculated 24 hours later with 5, 10 and 20 μl of VSV-G/HIV-1/Luc virus in triplicate. Cells were lysed 48 hours after virus inoculation and luciferase activity in the lysates was measured. HIV-1 infectivity is represented as average fold increase or decrease in luciferase activity relative to control Siren-ve (S-ve) cells (A) or cells transfected with non-specific siRNA (B).

We next evaluated the effect of the BTBD1 and BTBD2 shRNA plasmids transfected individually on permissiveness to HIV-1 infection in COS-1 cells. Cells transfected with the BTBD2 shRNA plasmid were 2.5 to 3-fold more permissive to HIV-1 infection (Figure 3A). In contrast, transfection of BTBD1 shRNA did not increase permissiveness to infection (data not shown). For the sake of comparison, we used the siRNA mediated approach to deplete TRIM5α in COS-1 cells as done by others [8,21-23]. Similar to their results, siRNA targeting TRIM5 increased permissiveness to HIV-1 infection up to 20 fold (Figure 3B), which is roughly eight-fold more permissiveness than we observed in AGM cells transfected with the BTBD2 shRNA plasmid.

## Discussion

**Figure 4:**
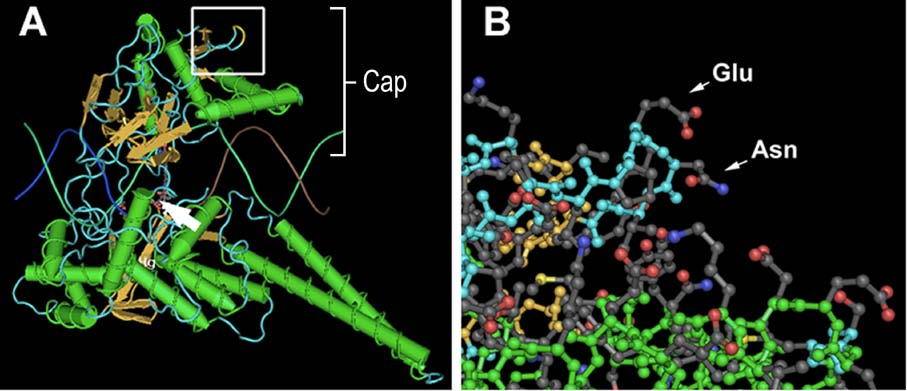
***hu*-TOP1 residues E236/N237 in crystal structure**. ***A***. Structure of TOP1 in covalent complex with topotecan (MMDB: 49892) shows active site (arrow), the cap region (core subdomains I and II, [20]), and residues E236 and N237 (yellow) within the box. ***B***. Enlargement of boxed region in panel-A shows that E236 and N237 are solvent exposed.

Shoya et al. reported that expression of *hu*-TOP1, but not AGM-TOP1, in the AGM producer cells increased infectivity of the progeny virus five-fold [7]. Only eight amino acid residues differ between AGM-TOP1 and *hu*-TOP1. Substitution of the human residues with the AGM residues was used to identify the human residues required for the infectivity enhancement. Only replacement of glutamate-236 and asparagine-237 of *hu*-TOP1 with the corresponding conserved monkey residues (aspartate and serine, respectively) abolished the infectivity enhancement. E236 and N237 of *hu*-TOP1 are far from the active site (Figure 4), solvent exposed, and their replacement with the AGM counterparts (E236D/N237S) did not affect DNA relaxation activity (data not shown).

Shoya et al. reported that the enhanced infectivity of HIV-1 virions, provided by expression of *hu*-TOP1, but not AGM-TOP1 or E236D/N237S-*hu*-TOP1, was not a consequence of the amount of virus produced, the processing of Gag or Env, nor viral entry, but was associated with a four-fold greater copy number of HIV-1 DNA in human target cells. They additionally showed expression of AGM-TOP1 or E236D/N237S-*hu*-TOP1 in human virion producer cells acts in a dominant negative manner to reduce the infectivity of progeny virions [7].

E236 and N237 are located within a fragment of *hu*-TOP1 comprising 15% of the protein previously shown to be required for binding BTBD1 and BTBD2. Here we have shown that *hu*-TOP1 with replacements of E236 and N237 with the AGM residues D236 and S237 did not bind BTBD1 or BTBD2 in two-hybrid and GST-pulldown assays. Thus, the two residues required for *hu*-TOP1 expression in AGM producer cells to enhance the infectivity of HIV-1 virions are also required for the interaction with BTBD1 and BTBD2, two proteins which co-localize with TRIM5γ (unpublished) and TRIM5δ [13].

We also found that depletion of BTBD2 in COS-1 cells increased their permissiveness to HIV-1 infection by 2.5 to 3-fold. Similarly, in human embryonic kidney 293T cells, depletion of BTBD2 increased permissiveness to infection by 1.8 to 2.8-fold (data not shown). For comparison, TRIM5α of AGM strongly restricts HIV-1 infection [8,24,25] (~20-fold in our experiments, Figure 3B), while human TRIM5α only modestly inhibits HIV-1 infection by about 50% [8,25]. Our data show that in both human and AGM cells, depletion of BTBD2 only modestly increased permissiveness to HIV-1 infection by about 2-3 fold. Thus, this modest restriction involving BTBD2 did not require the high level restriction provided by TRIM5α of Old World primates.

BTBD1 and BTBD2 localize in cytoplasmic bodies with each other [14] and interact with each other in two hybrid assays (unpublished results). BTBD1 binds Cul3 in co-precipitation assays using bacterially purified proteins, and flag-tagged BTBD1 bound the Cul3 N-terminal 197 aa in transfected 293T cells [15]. Cul-based and other ubiquitin E3 ligases catalyze ubiquitin transfer to substrate proteins by positioning their lysines for optimal presentation to ubiquitin bound to the E2 active site [26]. Some BTB domain proteins are involved in selecting substrates for Cul3 ubiquitin ligases [15-18].

Our data do not rule out the speculation that the increased permissiveness to HIV-1 infection resulting from BTBD2 silencing might in some way relate to the co-localization of BTBD1 and BTBD2 with TRIM5 proteins in cytoplasmic bodies or the involvement of BTBD1 and BTBD2 with other factors that may influence permissiveness to HIV-1 infection [25]. BTBD1 and BTBD2 co-localize with TRIM5δ and TRIM5γ, but these variants lack the C-terminal B30.2 domain that interacts directly with the viral capsid and determines specificity for virus restriction [27-29]. TRIM5γ and TRIM5δ, possibly by hetero-multimerizing with TRIM5α, can act dominant-negatively to inhibit the viral restricting activity of TRIM5α [8,30].

## Conclusions

The results presented here demonstrate that the interaction of BTBD1 and BTBD2 with TOP1 requires residues 236 and 237 of *hu*-TOP1, the same residues required for *hu*-TOP1 when expressed in AGM virion producer cells to enhance the infectivity of progeny virions. Interference with the expression of BTBD2 in either AGM or human cells increased their permissiveness to HIV-1 infectivity two- to three-fold.

## Competing interests

The authors declare that they have no competing interests.

## Authors' contributions

B.K. carried out shRNA knockdown, immunofluorescence and infectivity studies, and drafted portions of the manuscript. L.Z. and P.M. prepared mutant Top1 and carried out binding studies. T.S. prepared and provided VSV-G pseudotyped NL4-3 Luciferase virus particles. T.S. and C.B. provided HIV vectors, advice and consultation on infectivity assays, and edited the manuscript. M.L.C. and P.D. directed the study and wrote the manuscript. All authors read and approved the final manuscript.
